# Subsidence following cervical discectomy and implant-to-bone ratio

**DOI:** 10.1186/s12891-022-05698-8

**Published:** 2022-08-04

**Authors:** Bartosz Godlewski, Adam Bebenek, Maciej Dominiak, Grzegorz Karpinski, Piotr Cieslik, Tomasz Pawelczyk

**Affiliations:** 1 Department of Orthopaedics and Traumatology, With Spinal Surgery Ward. Scanmed St. Raphael Hospital, ul. Adama Bochenka 12, 30-693 Cracow, Poland; 2 Faculty of Medicine, Jagiellonian University Medical College, Cracow, Poland; 3 Department of Orthopaedics and Traumatology, Military Institute of Medicine, Warsaw, Poland; 4 Department of Affective and Psychotic Disorders, Medical University of Lodz, Lodz, Poland

**Keywords:** Subsidence, Radiological measurements, Cage size, Polyetheretherketone (PEEK), Titanium-coated PEEK

## Abstract

**Background:**

Implant subsidence is an undesirable effect after anterior cervical discectomy and fusion (ACDF). We investigated the relation between the rate of implant subsidence and the ratio of the implant surface area to the surface area of the adjacent bone.

**Methods:**

We operated 170 disc spaces in a group of 104 patients. Two types of implants were used: 1) PEEK (polyetheretherketone) cages and 2) titanium-coated (TC) PEEK cages. Patients were randomised to receive a specific implant using a randomisation table. All implants had a surface area of 1.61 cm^2^. Based on computed tomography images, bone surface areas were calculated for vertebral bodies immediately adjacent to the interbody implants. The implant-to-bone surface ratio was then calculated for each disc space. Implant subsidence was assessed over 12 months of follow-up, and associations between implant subsidence, the type of implant, and the implant-to-bone surface ratio were investigated.

**Results:**

Twelve months after the surgery, computed tomography was performed on 86 patients (144 disc spaces). Furthermore, in 166 disc spaces and 102 patients, conventional radiographs were obtained.

Subsidence was observed in 21% of the examined intervertebral spaces, and it was more frequently associated with higher values of bone surface area and lower values of the implant-to-bone surface ratio. The type of implant (PEEK vs TC-PEEK cages) did not significantly influence the rate of implant subsidence.

**Conclusions:**

Implant subsidence was significantly related to the value of a coefficient representing the ratio of the implant's surface area to the bone surface area of the adjacent vertebral bodies, with subsidence occurring significantly more rarely for coefficient values ≥ 0.37.

## Background

Cervical discectomy and fusion is the prevailing procedure performed for degenerative cervical disease. Cervical disc replacement with a stand-alone cage can restore physiologic disc height, provide immediate load-bearing support to the cervical spine and may promote osseous fusion. Implant subsidence after ACDF is an undesirable effect that should be prevented [1–5]. We investigated the relation between implant subsidence and the ratio of the surface area of the implant to that of the adjacent bone.

## Methods

**Fig. 1:**
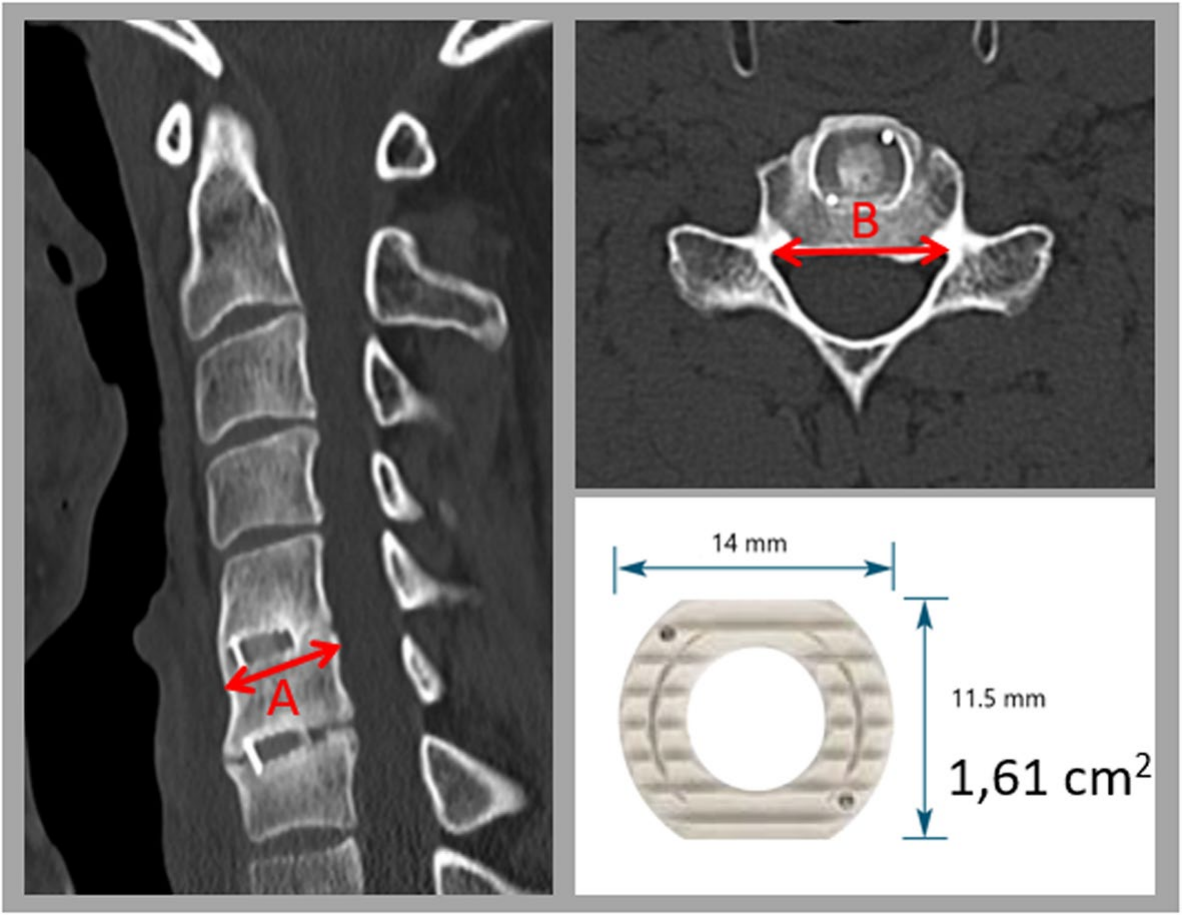
Procedure for measuring bone surface area of vertebral bodies immediately adjacent to implants on the basis of CT images obtained at 12 months post-surgery. **A** (cm) x **B** (cm) = surface area (cm^2^). All implants had the same surface area of 1.61 cm.^2^

**Fig. 2:**
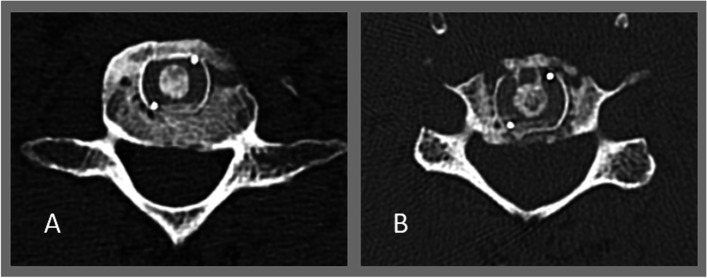
Comparison of the implant/bone surface ratio on the basis of two disc spaces. **A**) Patient No. 4, space No. 7, bone surface area of 4.62 cm^2^, implant surface area of 1.61 cm^2^, implant/bone ratio of 0.34. **B**) Patient No. 14, space No. 23, bone surface area of 3.68 cm^2^, implant surface area of 1.61 cm.^2^, implant/bone ratio of 0.43

**Fig. 3:**
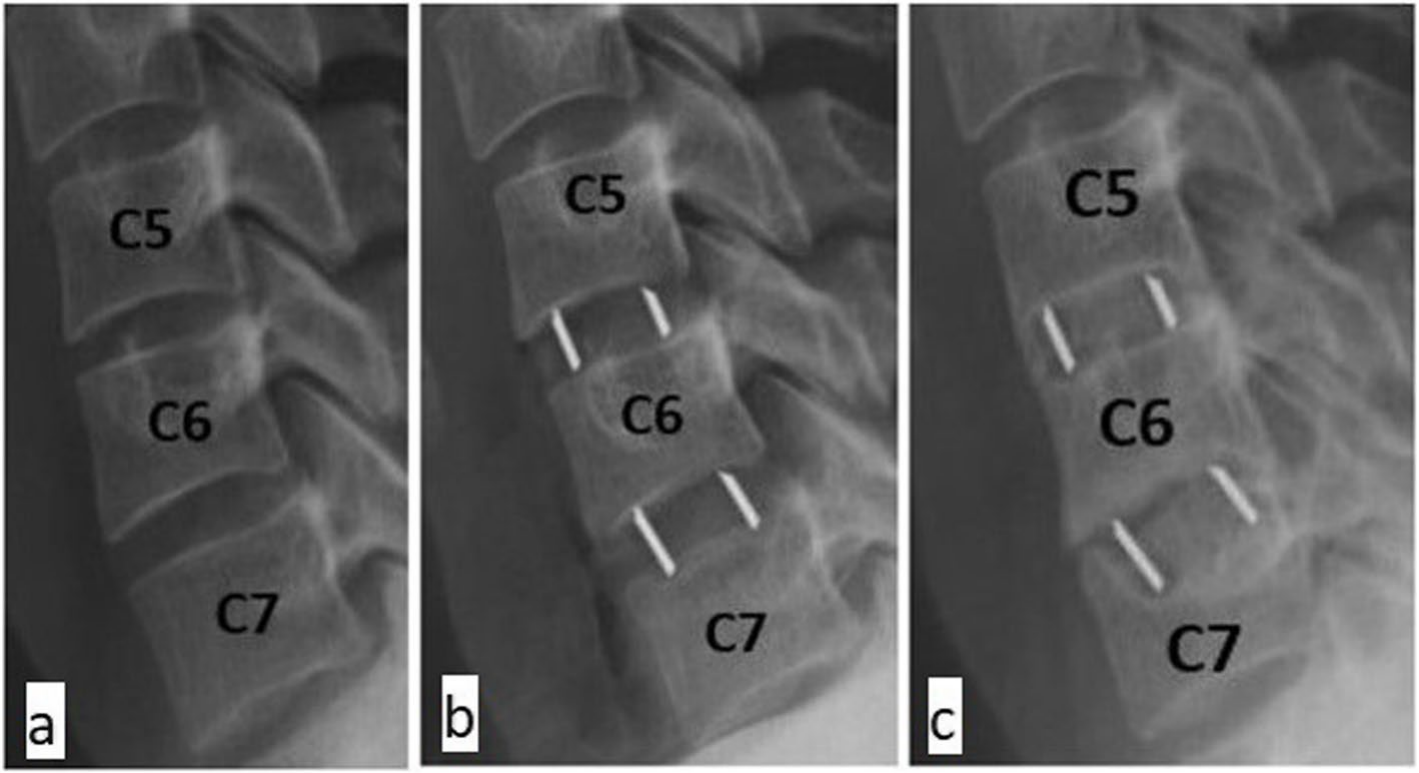
Example of cage subsidence. **a**) before surgery, **b**) one day after surgery, **c**) 12 months after surgery

We operated a total of 170 disc spaces in a group of 104 patients (age: 51.2 ± 10.3; female 73.1%). We used two types of implants: 1) PEEK (polyetheretherketone) cages and 2) titanium-coated (TC) PEEK cages. A randomisation table was used to assign patients a specific implant. Either one or two disc spaces were operated on during one surgery. All patients were operated on by the same surgeon and according to the same technique. In each case, the interior of an implant was filled with nanoparticle hydroxyapatite. Bone surface areas were calculated for vertebral bodies immediately adjacent to the interbody implants on the basis of computed tomography images. All implants used had the same surface area of 1.61 cm^2^ (Fig. 1). The implant-to-bone surface ratio was then calculated for each disc space (Fig. 2). Implant subsidence was assessed over 12 months of follow-up. We measured the height of interbody spaces in the centre of vertebral bodies, determining the distance between the endplates of adjacent vertebral bodies. The measurements were noted down with an accuracy of 1 mm. Measurements were made on radiographs obtained in one X-ray centre, following the same procedure and utilising the same equipment. The radiographic indices were evaluated at the following times: 1) before surgical procedure, 2) one day after surgery, 3) one month following the surgery, 4) six months post-surgery, 5) one year after the surgery. Subsidence was diagnosed if the implant displaced ≥ 3 mm into the adjacent endplates in relation to radiographs collected one day after the surgery (Fig. 3). Associations between implant subsidence and the type of implant as well as the coefficient representing the ratio of the implant surface area to the bone surface area of the adjacent vertebral bodies were investigated. The research was approved by the Bioethics Committee of the Andrzej Frycz Modrzewski University in Cracow (Resolution 4/2019) and was conducted in compliance with the Declaration of Helsinki.

## Results

**Fig. 4:**
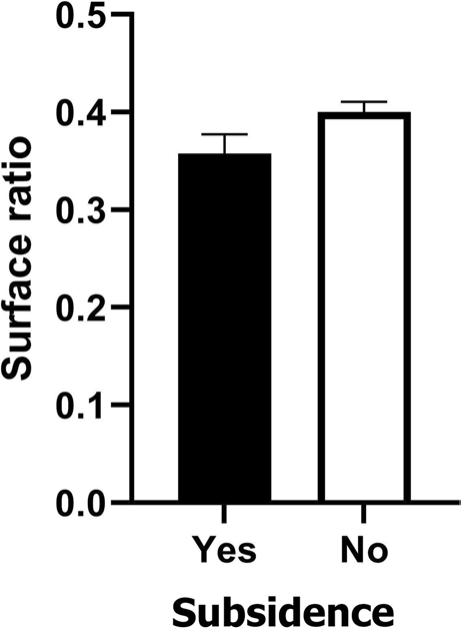
Differences in surface ratio between cases where subsidence was observed after 12 months (Yes) and those without subsidence (No). Bars show means ± 95% confidence Intervals

**Fig. 5:**
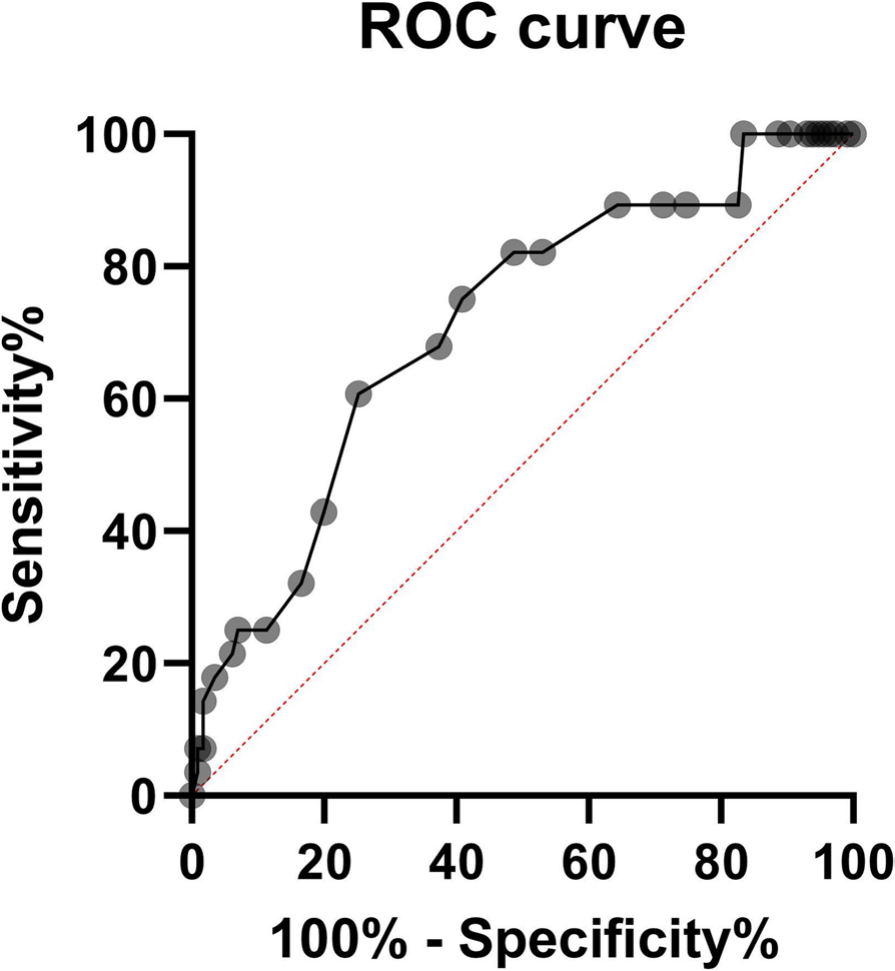
Receiver Operating Characteristics (ROR) for the surface ratio and the presence of subsidence after 12 months. Area under curve, AUC = 0.71 (95% CI 0.606–0.815). Cut-off point was determined using Youden’s J statistic, which maximises sensitivity and specificity

We completed 104 surgical procedures, operating 170 disc spaces. A total of 100 PEEK implants and 70 TC-PEEK implants were used. No association was observed between subsidence and sex (Chi-squared = 0.708, df = 1, *p* = 0.4) or implant type used (Chi-squared = 0.501, df = 1, *p* = 0.479). No significant associations were seen between age and subsidence status (Student’s t = 1,28, df = 99, *p* = 0,203). Significant correlations were observed between age and vertebral surface area (Pearson’s *r* = 0.229, *p* = 0.0352) and implant-to-bone surface area ratio (Pearson’s *r* = -0.222, *p* = 0.0412). Twelve months following the surgery, CT scans were performed on 86 patients (144 disc spaces), while typical radiographs were done on 102 patients (166 disc spaces). 166 complete sets of radiographic measurement data were used to assess changes in interbody space height and the presence of implant subsidence (98 for PEEK cages and 68 for TC-PEEK cages). Subsidence was identified in 21% of cases (35 disc spaces), which included 21 PEEK cages (21.4%) and 14 TC-PEEK cages (20.6%). Statistical analysis failed to find a significant association between the type of implant (PEEK vs TC-PEEK) and the presence of implant subsidence (Chi-squared 0.017, df = 1, *p* = 0.89615) (Table 1). Linear regression was used to assess the association between bone surface measurements and subsidence. Since there was a significant correlation between age and bone surface area and implant-to-bone surface area ratio, age was used in analyses as a covariate to control the effect of age on the results. Subsidence was significantly more frequently associated with a) higher values of bone surface area and b) lower values of the implant-to-bone surface area ratio (linear regression: a) *R*^2^ = 0.189; B = 0.997; *p* = 0.0004; b) *R*^2^ = 0.15; B = -0.057; *p* = 0.0025) (Table 2; Fig. 4). Analysis of the Receiver Operating Characteristics (ROC) curve and Youden’s J statistic [6, 7] calculated on its basis served to determine the value of the coefficient representing the ratio of an implant to vertebral body surface area that significantly differentiated the rate of implant subsidence, at ≥ 0.37 (Fig. 5). There was a correlation between the incidence of implant subsidence and the level treated, with subsidence seen most often following surgery at the C6/C7 level (51%), and least often following surgery at the C3/C4 level (3%). Implant subsidence was noted at 12 months post-surgery in 34.6% of all cases of C6/C7 surgery and 11.8% of all cases at the C3/C4 level (Chi-squared 8.502, df = 3, *p* = 0.0367). These results are summarised in Table 3.

**Table 1:** Association between type of implant and subsidence rate at 12 months post-surgery

Type of implant	Subsidence		χ^2^ (df)	p
	**Yes** **n (%)**^a^	**No** **n (%)**^a^		
**Titanium-coated PEEK**	14 (40)	54 (41)	0.017 (1)	0.8962
**PEEK**	21 (60)	77 (59)		

Key to abbreviations and symbols:

^a^column percentages;

*Χ*^*2*^ value of the statistic of the Chi2 test for independence, *df* Degrees of freedom, *p* Two-tailed test probability for Chi2 test statistics;

**Table 2:** Relationships between bone surface and implant-to-bone surface area ratio and subsidence, including age as a covariate to control for the confounding variable

Parameter	Subsidence Marginal mean (SE)		B	SE	95% CI for B	t	p
	**No**	**Yes**					
**Bone Surface**^**a**^	**3.93 (0.065)**	**4.53 (0.147)**	**0.597**	**0.161**	**(0.278-0.197)**	**3.72**	**0.0004**
**Implant-to-bone surface ratio area**^**b**^	**0.412 (0.0066)**	**0.361 (0.0149)**	**- 0.05**	**0.0164**	**(-0.084 --0.019)**	**-3.12**	**0.0025**

*Key to abbreviations and symbols:*

^a^R^2^ for the model 0.189;

^b^R^2^ for the model 0.15;

*B* linear regression coefficient estimate, *SE* Standard error of the estimate, *CI* Confidence interval, *t* t statistics for the linear regression, *p* two-tailed test probability for t-test statistic;

Underline marks significant associations for *p* < α = 0.05

**Table 3:** Implant subsidence rate and treatment level

Treatment level	Subsidence		χ^2^ (df)	p
	**Yes** **n (column %) row %**	**No** **n (column %) row %**		
C3/C4	1 (2.9) 11.11	8 (6.11) 88.89	8.502 (1)	**0.0367**
C4/C5	2 (5.71) 12.5	14 (10.7) 87.5		
C5/C6	14 (40.0) 15.73	75 (57.3) 84.27		
C6/C7	18 (51.43) 34.62	34 (25.95) 65.38		

Key to abbreviations and symbols:

*Χ*^*2*^ value of the statistic of the Chi2 test for independence, *df* degrees of freedom, *p* two-tailed test probability for Chi2 test statistics;

Underline marks significant associations for *p* < α = 0.05; α – level of statistical significance; sample size differences are due to missing data

## Discussion

Most cervical spine procedures in patients with degenerative disc disease involve discectomy and removal of osteophytes in posterior vertebral body surfaces followed by interbody stabilisation with an interbody implant (cage). Cages are made of a variety of materials, differing in structural design, shape and surface topography. Implant subsidence is a widely known and recognised phenomenon. A number of definitions of implant subsidence exist. It may be defined as the length of immersion of the implant (in millimetres) beyond the borders of the adjacent endplates or as the percentage reduction in interbody space height [8–12]. Cage subsidence may influence spinal biomechanics and alignment, cause segmental kyphosis and contribute to adjacent segment disease. The decreased height of the interbody space may lead to foraminal stenosis [2–4, 10–14]. Current literature suggests a subsidence rate ranging from 19.3 to 42.5% [1, 12]. There is currently controversy and debate on the correlation between cage subsidence and clinical outcome [1, 3, 8, 10–17]. We investigated implants made of PEEK and TC-PEEK. The elasticity modulus of PEEK is similar to that of bone, which results in minimising subsidence and optimising the interaction of the compressive forces at the graft-host interface. Since PEEK implants do not distort the anatomical image, they are advantageous in subsequent post-operative imaging. The radiolucent property of PEEK allows for the appropriate assessment of bone in-growth. Furthermore, compared to metallic implants, PEEK produces fewer artefacts on MRI and CT scans [2–20]. TC-PEEK cages preserve the biomechanical and radiographic advantages of PEEK. Improved osseointegration is achieved by adding a plasma-sprayed surface layer of titanium. Our statistical analyses showed that the type of implant (PEEK vs TC-PEEK) did not significantly affect implant subsidence. A significant association was detected between the rate of implant subsidence and the ratio of the implant surface area to the bone surface area of the adjacent vertebral bodies. Some papers show that anterior implant placement within the disk space reduces the risk of subsidence compared to more posteriorly placed implants [1–3, 21, 22]. Park J-Y et al. found that a distance of ≥ 3 mm between the anterior margin of the vertebral body and that of the cage was a statistically significant risk factor for subsidence (*p* < 0.001) [2]. Some papers indicate that implant subsidence can be limited with additional fixation with a cervical plate. Instrumentation appears to be helpful, particularly for ACDF involving two or more levels [23]. Dai and Jiang studied radiologic and clinical results after ACDF with and without a plate and reported higher subsidence in the ACDF group without a plate [24]. The rate of subsidence is also influenced by implant size and the size of the adjacent endplates between which the implant is placed. Implant size should match the specific size of the adjacent endplates to reduce the risk of subsidence [1, 3, 21, 25]. Smaller cages may be more prone to subsidence on account of the smaller area to distribute the acting forces, and the absence of support on harder bone found at the edges of vertebral endplates [1, 25–28, 29, 30]. Mende KS et al. observed a significant correlation between the cage/endplate ratio and the incidence of subsidence. Their measurements were based on radiographs. Cages covering more than 65% of the sagittal endplate diameter were significantly less frequent to subside than those below 65% (overall: 64.6 vs 35.4%, *p* < 0.01) [1]. Yang et al. demonstrated that a small anterior–posterior cage diameter (12 mm vs 14 mm) and a large intraoperative distraction significantly increased the risk of subsidence [31]. In our study, we calculated surface areas of the vertebrae immediately adjacent to the interbody implants on the basis of CT images. The ratio of implant surface area to bone surface area was computed for each interbody space analysed. We established that implant subsidence occurred significantly less often when the coefficient was ≥ 0.37. We also observed a correlation between the subsidence rate and the treatment level, with subsidence occurring significantly more frequently after surgery at the level of C5/C6 and C6/C7 than C3/C4 or C4/C5 (Table 3) (Pearson’s Chi^2, Chi-squared 8.501849, df = 3, *p* = 0.03670). Similar results were obtained by Kao et al., who mentioned that the treatment levels below the C5 level (C5/C6, C6/C7) had more chance of subsidence than treatment levels above the C5 level (C2/C3, C3/C4, C4/C5) [19]. Bartels et al. reported that the incidence of cage subsidence was significantly higher for C6/C7 fusions than for fusions at other levels  [32]. With regard to pathophysiology, some of the endplates should be removed to expose the subchondral bone to facilitate fusion. The use of a high-speed drill can facilitate this, but surgeons need to pay close attention to endplate integrity in order to prevent subsidence. In our experience, in most cases, the disc and osteophytes of posterior vertebral body edges can be removed, and sufficient decompression of neural structures can be performed without disrupting endplate continuity.

## Conclusions

The type of implant (PEEK vs titanium-coated PEEK cages) did not significantly affect the rate of implant subsidence. Subsidence was significantly associated with the ratio of the implant surface area to the surface area of the adjacent vertebral bodies, with subsidence being much less frequent for ratios ≥ 0.37. 

## Data Availability

The datasets used and /or analysed during the current study are available from the corresponding author on reasonable request.

## Abbreviations

CT: Computed Tomography
PEEK: Polyetheretherketone
ROC: Receiver Operating Characteristics
TC-PEEK: Titanium-coated PEEK
